# The effects of music listening intervention on postoperative care in unilateral vs. bilateral endometriotic cysts

**DOI:** 10.3389/fmed.2025.1626575

**Published:** 2025-09-15

**Authors:** Xiaohui Yang, Yue Meng, Huiyan Feng, Ya Long, Zhijia Zheng, Xiaomao Li, Qingjian Ye

**Affiliations:** Department of Gynecology, The Third Affiliated Hospital of Sun Yat-sen University, Guangzhou, China

**Keywords:** endometriosis, music listening intervention, pain, anxiety, depression, fatigue

## Abstract

**Background:**

Endometriosis is a chronic gynecological condition that can result in pelvic pain and infertility, thereby significantly impacting women's reproductive health. Psychological disorders such as anxiety and depression are common in women with endometriosis and can have a detrimental effect on their quality of life. Conventional postoperative care primarily focuses on medical and surgical aspects; however, there is a growing need to address the psychological wellbeing of patients as part of comprehensive postoperative care. Music listening interventions have emerged as a promising adjunctive approach due to their non-invasive and non-pharmacological nature, which can potentially enhance the postoperative experience by targeting both physical and psychological aspects.

**Methods:**

This study is a secondary analysis of a randomized controlled trial with 141 young nulliparous participants who underwent laparoscopic endometriosis cyst resection at Sun Yat-sen University's Third Affiliated Hospital from September 2023 to August 2024. Participants were divided into a music listening group (*n* = 72) and a control group (*n* = 69). The music listening group received 30-min daily sessions starting the day before surgery for 7 days.

**Results:**

141 patients were categorized into two groups: the unilateral cyst group (93 patients) and the bilateral cyst group (48 patients). In both unilateral and bilateral cyst cohorts, the intervention failed to demonstrate efficacy in reducing postoperative nausea/vomiting or pain intensity (all *P* > 0.05). Similarly, anxiety (GAD-7), depression (PHQ-9), and fatigue (FSS) scores showed no significant improvements in either subgroup at any postoperative assessment (all *P* > 0.05). A transient reduction in anxiety (GAD-7) was observed in bilateral cyst patients at day 7 (*P* = 0.046), but this effect lost significance after Bonferroni correction (adj*P* = 0.460). Longitudinal changes in psychological scores from baseline to postoperative days 1–7 were also non-significant.

**Conclusion:**

This secondary analysis indicates that a 7-day music listening intervention (30 min/day) doesn't confer significant benefits for postoperative pain, nausea/vomiting, anxiety, depression, or fatigue in young nulliparous patients undergoing laparoscopic endometriotic cystectomy, irrespective of unilateral or bilateral cyst involvement. Future studies should investigate optimized protocols (e.g., therapist-guided sessions, longer duration) and mechanistic biomarkers to identify potential responders.

## 1 Introduction

Endometriosis, affecting about 10% of women and uterus-bearing individuals worldwide, often causes pelvic pain and infertility (1–3). It also heightens the risk of mental health issues like anxiety and depression, reducing quality of life (4–6). Endometriotic cysts represent a prevalent manifestation of endometriosis, with standard treatment modalities encompassing pharmacological intervention and surgical procedures. Nevertheless, individuals undergoing surgical treatment are predisposed to an increased incidence of adverse psychological outcomes, such as anxiety and depression (6, 7). These psychological sequelae not only impede postoperative recovery but may also exert enduring detrimental impacts on mental health and overall quality of life (8).

Traditional postoperative care primarily addresses medical and surgical needs; however, there is an increasing awareness of the importance of psychological wellbeing. Music listening intervention, a non-drug intervention, is gaining attention for its therapeutic benefits, particularly in pain management. Studies show that music can effectively reduce pain intensity (9, 10). Listening to music after surgery can lessen pain catastrophizing and may also enhance psychological and physical wellbeing during negative pain experiences (10). In the context of anxiety management, music has been empirically validated as a potent therapeutic intervention (10–12). Music distracts patients during medical procedures and reduces their perception of pain and anxiety-inducing stimuli. At the same time, it lowers the excitability of the sympathetic nervous system, slows down heart rate, and lowers blood pressure, thereby reducing anxiety (13). Research indicates that permitting patients undergoing gynecological pelvic reconstruction to listen to their preferred music on the day of the procedure constitutes a straightforward intervention that can effectively alleviate preoperative anxiety and enhance patient satisfaction (14).

However, the effect of music listening intervention in a specific subgroup of patients with endometriosis has not been fully explored. Our primary randomized controlled trial (RCT) in this study found that music intervention had a limited impact on perioperative anxiety and postoperative pain in young nulliparous patients who underwent laparoscopic endometriosis cystectomy (15). Research indicates that bilateral ovarian endometriotic cysts cause more harm to ovarian reserve than unilateral ones. Before surgery, patients with bilateral cysts have significantly lower anti-Mullerian hormone (AMH) levels (16), which continue to decline post-operation, unlike those with unilateral cysts, whose levels partially recover (17, 18). This disparity may heighten fertility concerns and increase perioperative anxiety and depression, making them key targets for our music listening intervention. Additionally, bilateral cysts are often linked to advanced endometriosis and severe adhesions (19). Extensive adhesions prolong the operation time, increase the risk of postoperative pain, and may potentially affect patients' responses to non-pharmacological interventions such as music listening intervention. In light of this, we further focus on the impact of music listening intervention on these symptoms. Furthermore, studies have shown that listening to music can alleviate the severity of depressive symptoms and improve sleep quality (20, 21). Therefore, in addition to examining the main outcomes related to pain, anxiety, and postoperative nausea and vomiting, we also included data on depression and fatigue to provide a more comprehensive assessment of the impact of the intervention on postoperative care for this patient group.

Given the current lack of evidence on music listening intervention, especially in this patient population, this secondary analysis adopts an exploratory approach. The main exploratory objective was to compare the effects of a 7-day music intervention and standard care alone on postoperative nausea, vomiting, pain intensity, postoperative anxiety, depression, and fatigue in young nulliparous patients with unilateral and bilateral endometriotic cysts who underwent laparoscopic endometriotic cystectomy. We hypothesized that patients' responses to music listening intervention might vary depending on the laterality of the cysts. This variation could be due to different degrees of disease burden and psychological vulnerability. In particular, patients with bilateral cysts face greater fertility issues and surgical complexity and may benefit more from the emotional and psychological support provided by music intervention.

## 2 Materials and methods

### 2.1 Study population

This study represents a secondary exploratory analysis of an RCT investigating music intervention in endometriosis cystectomy patients (15). In our primary RCT survey, a cohort of young, 141 nulliparous patients who underwent laparoscopic endometriotic cystectomy at the Third Affiliated Hospital of Sun Yat-sen University from September 2023 to August 2024 was assembled. This research was a randomized controlled trial and has been ethically approved by the Third Affiliated Hospital of Sun Yat-Sen University (II2023-292-02).

The inclusion criteria were as follows: (1) patients undergoing laparoscopic endometriotic cystectomy; (2) aged 20–35 years, without previous childbirth, and with fertility intentions; (3) willingness to adhere to the prescribed medical treatment plan and participate in follow-up assessments; and (4) provision of informed consent by all participants. The exclusion criteria included: (1) the presence of malignant tumors; (2) presence of psychiatric and/or cognitive disorders that prevent understanding of the study's purpose or compliance with medical advice; (3) those with an aversion to music; (4) individuals experiencing other chronic pain conditions; and (5) those unwilling to participate or who withdrew from the study.

Sample size estimation was performed using an online tool named POWER AND SAMPLE SIZE (http://powerandsamplesize.com/Calculators/), setting parameters based on similar studies: a Type I error rate of 0.1, power of 0.8, true proportion of 0.5, null hypothesis proportion of 0.3, and a 20% attrition rate. This calculated a needed sample size of 44 per group, totaling 88.

**Figure 1:**
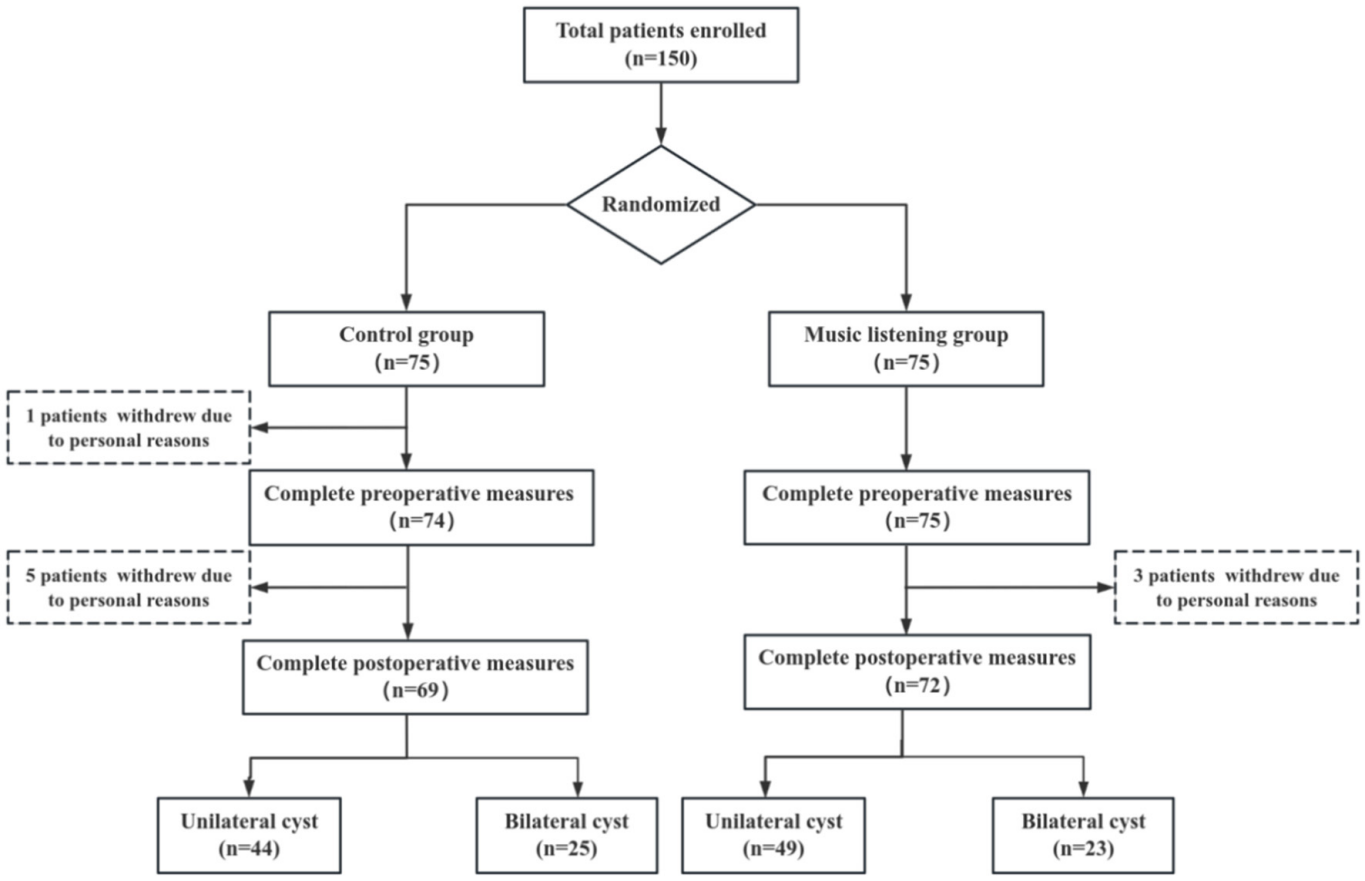
Flow diagram for the study.

At the commencement of the study, a total of 150 patients were enrolled and subjected to random allocation. Statisticians employed the SPSS 25.0 random number table to generate random numbers, facilitating the division of participants into two groups: the music listening group (*N* = 75) and the control group (*N* = 75). A CONSORT flow diagram (Figure 1) detailing participant flow, including reasons for withdrawal, is provided in the Results section. The final analysis included 72 participants in the music listening group and 69 participants in the control group.

### 2.2 Preoperative measures

Before surgery, patient demographics (age, marital status, height, and weight) were collected. Preoperative pain, anxiety, depression, and stress were assessed using:

(1) Visual Analog Scale (VAS) for pain intensity (0–10; 0 = no pain, 10 = severe pain).
(2) Generalized Anxiety Disorder Scale (GAD-7) for anxiety. Each item is scored 0–3, with total scores ranging 0–21 (0–4 = minimal, 5–9 = mild, 10–14 = moderate, 15–21 = severe anxiety).
(3) Patient Health Questionnaire-9 (PHQ-9) for depression. Each item is scored 0–3, with total scores ranging 0–27 (0–4 = minimal, 5–9 = mild, 10–14 = moderate, 15–19 = moderately severe, 20–27 = severe depression).
(4) Fatigue Severity Scale (FSS) for fatigue (score < 36 = no significant fatigue, ≥36 = fatigue present).

### 2.3 Intervention and control groups

All participants underwent laparoscopic cystectomy for endometriotic ovarian cysts and received standard perioperative care. The intervention group was provided with an adjunctive music listening intervention in addition to the standard care, whereas the control group received only the standard care.

In the intervention group, patients independently selected music through the “Music for Emotional Regulation” module within the “Cloud SanYuan” WeChat mini-program of the Third Affiliated Hospital of Sun Yat-sen University. This mini-program was developed by professional music therapists. The music library within the program is categorized based on therapeutic functions, emotional attributes, and musical characteristics, all defined according to principles of music therapy and clinical experience. The categories include:

(1) Therapeutic function-based classifications: anxiety- relieving, depression-alleviating, and hypnotic music;
(2) Emotion-or imagery-based classifications: emotional resonance, excitement, lightness, passion, quietness, beauty, and cheerfulness;
(3) Acoustic characteristic-based classifications: low pitch, narration, and softness.

This therapeutic intervention began preoperatively (Day 1) and continued for 7 days postoperatively, consisting of one 30-min session each day to complete the full therapeutic course. Patients in the music intervention group selected their music daily and listened to it at a comfortable volume for 30 min, using either speakers or headphones. Each participant recorded the time spent listening to music and the type of music they listened to daily. During hospitalization, professional nurses promptly collected the completed questionnaires. After discharge, participants were asked to continue listening to music at home. To enhance compliance and reduce dropout rates, research assistants contacted each participant daily by phone, recorded the details of their music listening, and assisted them in completing the questionnaire. The methods of music listening intervention and the quality control measures are detailed in our previously published primary study (15). A Quick Response (QR) code for accessing the music WeChat mini-program is available in Supplementary material 1.

### 2.4 Postoperative measures

Postoperative observation indicators included postoperative nausea, vomiting, postoperative pain (VAS score), anxiety (GAD-7 questionnaire), depression (PHQ-9 questionnaire), and fatigue (FSS questionnaire). These indicators were collected through the following methods:

(1) Assessment of Postoperative Nausea and Vomiting.
  Postoperative nausea and vomiting were assessed through direct patient interviews conducted by trained nursing staff at three time points: 2, 4, and 6 h following surgery. Patients were asked the following questions: (1) “1)s you currently experiencing nausea?” (yes/no) and (2) “2)s/ you vomited since the last assessment?” (yes/no). Immediately after obtaining the patient's response, the nurse recorded the answers and the corresponding assessment time on a pre-designed paper-and-pencil registration form. The paper forms were checked daily by the research coordinator to confirm no missing data or recording errors.
(2) Evaluation of Postoperative Pain.
  Postoperative pain intensity was evaluated using the Visual Analog Scale (VAS). VAS scores were collected at standardized time points: at 6 hours after surgery, and between 8:00 AM and 12:00 AM on postoperative days 1, 3, and 7. The nurse immediately recorded the reported VAS score, assessment time, and the patient's name/bed number on a dedicated paper-and-pencil VAS registration form. The form included a reminder to confirm the patient's understanding of the scale, and nurses were required to document any clarification provided to the patient (e.g., re-explaining the scale) to ensure transparency.
(3) Assessment of Anxiety, Depression, and Fatigue.
  The GAD-7, PHQ-9, and FSS scores were obtained on the 1st, 3rd, and 7th days following surgery to assess levels of anxiety, depression, and fatigue among the patients. All three questionnaires were administered uniformly via the WeChat “eChatmlyrede (Questionnaire Star) mini-program to ensure standardization of presentation and response format across all participants and time points.

### 2.5 Statistical analysis

Statistical analysis was performed using SPSS 27.0. Categorical variables were presented as frequencies and percentages, with group comparisons executed via the Chi-square test or Fisher's exact test. A Shapiro–Wilk test was employed to assess the normality of continuous variables. Variables adhering to a normal distribution were reported as mean ± standard deviation, and inter-group comparisons were performed using the independent samples Student's t-tests. Conversely, variables not following a normal distribution were described using the median and interquartile range (P25, P75), with the Mann-Whitney U test applied for inter-group comparisons.

Change scores were calculated as the difference between measurements at later time points and earlier time points [e.g., “Change (1 day to 6 h)” for pain was defined as VAS score at 1 day postoperatively minus VAS score at 6 hours postoperatively; “Change (3 day to 1 day)” for anxiety was defined as GAD-7 score at 3 days postoperatively minus GAD-7 score at 1 day postoperatively]. For change scores following a normal distribution, they were reported as mean ± standard deviation; for those not following a normal distribution, they were described using median and interquartile range (P25, P75), with statistical comparisons consistent with the methods used for the original variables (independent samples Student's *t*-tests for normally distributed change scores and Mann-Whitney U test for non-normally distributed ones).

To mitigate the risk of Type I errors due to multiple comparisons, a hierarchical Bonferroni correction was applied; further details can be found in the footnotes of **Tables 3, 4**. A two-tailed *P*-value less than the corresponding correction threshold was considered indicative of a statistically significant difference.

## 3 Results

### 3.1 Baseline characteristics

This study initially included 150 patients with endometriotic cysts. During the research process, three patients in the music intervention group and six patients in the control group withdrew due to personal reasons unrelated to the study. Therefore, a total of 141 patients were ultimately included for analysis, among whom 93 had unilateral cysts and 48 had bilateral cysts. For details, please refer to Figure 1.

In the preoperative assessment of psychological status, 60.99% of patients exhibited mild to severe anxiety, 53.90% demonstrated mild to severe depression, and 47.52% experienced fatigue. Statistical analysis revealed no significant differences in the levels of anxiety, depression, and fatigue between patients with unilateral and bilateral endometriosis cysts (Table 1). Statistical analysis revealed no significant differences in age, BMI, marital status, preoperative VAS scores, GAD-7scores, PHQ-9 scores, and FSS scores between the unilateral and bilateral cyst cohorts, nor between the control and music listening groups within each cyst type (Table 2).

**Table 1:** Preoperative psychological status of patients with endometriosis cysts.

**Psychological status**	**Total *N =* 141**	**Endometriosis cysts *N =* 141**		
		**Unilateral cyst (*****n** =* **93)**	**Bilateral cyst (*****n** =* **48)**	***P***
**Preoperative GAD-7**				0.112
Normal	55 (39.01%)	42 (45.16%)	13 (27.08%)	
Mild anxiety	72 (51.06%)	44 (47.31%)	28 (58.33%)	
Moderate anxiety	11 (7.80%)	5 (5.38%)	6 (12.50%)	
Severe anxiety	3 (2.13%)	2 (2.15%)	1 (2.08%)	
**Preoperative PHQ-9**				0.072
Normal	65 (46.10%)	48 (51.61%)	17 (35.42%)	
Mild depression	51 (36.17%)	27 (29.03%)	24 (50.00%)	
Moderate depression	22 (15.60%)	15 (16.13%)	7 (14.58%)	
Moderately severe depression	3 (2.13%)	3 (3.23%)	0 (0)	
**Preoperative FSS**				0.946
Normal	74 (52.48%)	49 (52.69%)	25 (52.08%)	
Fatigue	67 (47.52%)	44 (47.31%)	23 (47.92%)	

**Table 2:** Baseline characteristics of patients with unilateral and bilateral cyst.

**Characteristics**	**Unilateral cyst (*N =* 93)**		***P***	**Bilateral cyst (*N =* 48)**		***P***
	**Control (*****n** =* **44)**	**Music (*****n** =* **49)**		**Control (*****n** =* **25)**	**Music (*****n** =* **23)**	
Age	28.30 ± 3.76	28.22 ± 3.27	0.923	29.04 ± 2.57	29.52 ± 3.42	0.582
BMI	19.86 ± 2.88	20.45 ± 3.48	0.383	20.92 ± 3.35	19.78 ± 2.09	0.169
Marital status			0.127			0.556
Unmarried	35(79.55%)	32(65.31%)		15(60.00%)	16(69.57%)	
Married	9(20.45%)	17(34.69%)		10(40.00%)	7(30.43%)	
Preoperative VAS	2.00(1.00,4.00)	1.00(1.00,5.50)	0.826	4.00(1.00,6.50)	2.00(1.00,5.00)	0.311
Preoperative GAD-7	5.00(2.25,6.00)	5.00(2.00,7.00)	0.195	7.00(5.00,8.50)	6.00(4.00,8.00)	0.448
Preoperative PHQ-9	4.00(2.00,7.00)	5.00(2.00,9.00)	0.227	5.00(4.00,7.00)	7.00(3.00,9.00)	0.756
Preoperative FSS	35.68 ± 12.45	32.45 ± 10.61	0.180	34.40 ± 11.14	34.30 ± 12.36	0.978

### 3.2 Postoperative nausea, vomiting, and pain

Postoperative nausea and vomiting did not exhibit significant differences between the control and music listening groups at 2, 4, and 6 h following surgery in both unilateral and bilateral cyst cohorts (Table 3). Similarly, postoperative pain levels were not significantly different between the control and music listening groups at 6 hours, 1, 3, and 7 days post-surgery in both cyst groups. Furthermore, no statistically significant differences were observed in the change of postoperative pain from 6 h to 7 days post-surgery (Table 3).

**Table 3:** Postoperative nausea, vomiting, and pain of patients with unilateral and bilateral cysts.

**Characteristics**	**Unilateral cyst (*N =* 93)**		P^*^	**Bilateral cyst (*N =* 48)**		P^*^
	**Control (*****n** =* **44)**	**Music (*****n** =* **49)**		**Control (*****n** =* **25)**	**Music (*****n** =* **23)**	
**Postoperative nausea**						
Two hours postoperative			0.928			1.000^a^
Yes	6 (13.64%)	7 (14.29%)		3 (12.00%)	2 (8.70%)	
No	38 (86.36)	42 (85.71)		22 (88.00%)	21 (91.30%)	
Four hours postoperative			1.000^a^			0.235^a^
Yes	4 (9.09%)	4 (8.16%)		3 (12.00%)	0 (0)	
No	40 (90.91%)	45 (91.84%)		22 (88.00%)	23 (100%)	
Six hours postoperative			0.718^a^			1.000^a^
Yes	3 (6.82%)	5 (10.20%)		1 (4.00%)	1 (4.35%)	
No	41 (93.18%)	44 (89.80%)		24 (96.00%)	22 (95.65%)	
**Postoperative vomiting**						
Two hours postoperative			1.000^a^			1.000^a^
Yes	3 (6.82%)	4 (8.16%)		3 (12.00%)	2 (8.70%)	
No	41 (93.18%)	45 (91.84%)		22 (88.00%)	21 (91.30%)	
Four hours postoperative			0.496^a^			0.490^a^
Yes	0 (0)	2 (4.08%)		2 (8.00%)	0 (0)	
No	44 (100%)	47 (95.92%)		23 (92.00%)	23 (100%)	
Six hours postoperative			1.000^a^			1.000^a^
Yes	1 (2.27%)	1 (2.04%)		1 (4.00%)	1 (4.35%)	
No	43 (97.73%)	48 (97.96%)		24 (96.00%)	22 (95.65%)	
**Postoperative VAS**						
Six hours postoperative	2.00 (2.00,3.00)	3.00 (2.00,3.00)	0.522	2.00 (2.00,3.00)	2.00 (2.00,4.00)	0.139
One day postoperative	4.00 (3.00,6.00)	4.00 (3.00,5.00)	0.541	4.00 (3.00,7.00)	5.00(3.00,6.00)	0.684
Three days postoperative	2.00 (2.00,3.00)	3.00 (2.00,3.00)	0.800	2.00 (1.00,3.00)	2.00 (2.00,3.00)	0.867
Seven days postoperative	1.00(1.00,2.00)	2.00 (1.00,2.00)	0.666	1.00 (1.00,2.00)	1.00 (1.00,2.00)	0.593
Change (1 day to 6 hours)	1.50 (1.00,3.75)	1.00 (0,2.00)	0.370	2.00 (1.00,5.00)	2.00 (1.00,3.00)	0.165
Change (3 days to 6 hours)	0 (−1.00, 1.00)	0 (−1.00, 1.00)	0.795	−3.00 (−4.00, −0.50)	−2.00 (−3.00, −1.00)	0.645
Change (7 days to 6 hours)	−1.00 (−1.75, 0)	−1.00 (−2.00, 0)	0.808	−3.00 (−5.00, −1.50)	−4.00 (−4.00, −2.00)	0.826

^a^Fisher's exact test.

^*^After stratified Bonferroni correction: (1) For postoperative nausea (6 tests, α = 0.0083), all comparisons remained non-significant (smallest adjP = 1.000). (2) For postoperative vomiting (6 tests, α = 0.0083), no significant differences were observed (all adjP > 0.49). (3) For postoperative pain (VAS, 14 tests, α = 0.0036), no significant differences were observed (smallest rawP = 0.139, adjP = 1.000).

### 3.3 Postoperative anxiety, depression, and fatigue

Postoperative anxiety, as assessed by the GAD-7 scale, did not exhibit any significant differences between the control and music listening intervention groups among patients with unilateral cysts at any time point. In contrast, among patients with bilateral cysts, the music listening group demonstrated lower anxiety levels at 7 days post-operation (*P* = 0.046). However, this initially observed difference in GAD-7 scores at 7 days for bilateral cyst patients did not remain statistically significant following a stratified Bonferroni correction for multiple comparisons (adjusted *P* = 0.460). Furthermore, changes in GAD-7 scores over time were not significant for either type of cyst. Similarly, postoperative depression, evaluated using the PHQ-9, and fatigue, assessed by the FSS, did not show significant differences between the groups at any time point or over time for both unilateral and bilateral cyst patients (Table 4).

**Table 4:** Postoperative anxiety, depression, and fatigue of patients with unilateral and bilateral cysts.

**Characteristics**	**Unilateral cyst (*N =* 93)**		P^*^	**Bilateral cyst (*N =* 48)**		P^*^
	**Control (*****n** =* **44)**	**Music (*****n** =* **49)**		**Control (*****n** =* **25)**	**Music (*****n** =* **23)**	
**Postoperative GAD-7**						
One day postoperative	3.00 (1.00, 7.00)	5.00 (2.00, 7.00)	0.363	5.00 (1.50, 7.00)	5.00 (3.00, 9.00)	0.732
Three days postoperative	1.00 (0,4.00)	1.00 (0, 5.50)	0.984	0 (0, 4.00)	3.00 (0, 5.00)	0.076
Seven days postoperative	0 (0, 4.00)	0.50 (0, 5.75)	0.485	2.00 (0, 6.00)	0 (0, 4.00)	0.046
Change (3 days to 1 day)	−1.00 (−3.00, 0)	−1.00 (−2.50, 0)	0.516	−2.00 (−7.00, −0.50)	−2.50 (−5.25, 0)	0.640
Change (7 days to 1 day)	−2.00 (−5.00,0)	−2.00 (−5.00,1.00)	0.808	−2.00 (−5.50,−0.50)	−5.00 (−9.00,0)	0.199
**Postoperative PHQ-9**						
One day postoperative	5.00 (3.00, 9.00)	7.00 (3.00, 9.00)	0.250	4.00 (5.00, 8.00)	7.00 (4.00, 9.00)	0.493
Three days postoperative	3.00 (0, 5.00)	3.00 (0, 8.00)	0.723	3.00 (2.00, 6.00)	4.00 (2.00, 7.00)	0.442
Seven days postoperative	2.00 (0, 4.75)	1.00 (0, 6.00)	0.978	3.00 (1.00, 7.50)	1.00 (0, 4.00)	0.099
Change (3 days to 1 day)	−1.00 (−4.00, 0)	−2.00 (−4.00, −1.00)	0.078	−2.00 (−5.50, −0.50)	−2.00 (−5.00, 0)	0.950
Change (7 days to 1 day)	−3.00 (−5.00, −0.25)	−3.00 (−8.00, 0)	0.542	−3.00 (−5.00, −2.50)	−4.00 (−7.00, 0)	0.222
**Postoperative FSS**						
One day postoperative	25.61 ± 11.41	23.29 ± 11.29	0.326	24.52 ± 9.87	24.57 ± 12.30	0.989
Three days postoperative	23.61 ± 13.19	21.08 ± 12.50	0.345	23.32 ± 14.07	22.53 ± 13.21	0.841
Seven days postoperative	21.95 ± 10.69	21.27 ± 13.99	0.792	23.13 ± 13.40	22.00 ± 11.94	0.763
Change (3 days to 1 day)	−2.00 ± 8.39	−2.20 ± 6.70	0.897	−4.84 ± 6.90	−3.73 ± 6.96	0.515
Change (7 days to 1 day)	−3.66 ± 14.04	−2.02 ± 16.93	0.615	−7.28 ± 19.40	−6.65 ± 19.57	0.912

^*^After stratified Bonferroni correction (α = 0.005 per scale family): (1) For anxiety (GAD-7) (10 tests), all comparisons were non-significant (smallest adjP = 0.460 for bilateral cysts at 7 days). (2) For depression (PHQ-9) (10 tests), no significant differences were observed (smallest adjP = 0.780 for unilateral cysts in change scores). (3) For fatigue (FSS) (10 tests), music listening intervention showed no significant effects (all adjP > 0.326).

## 4 Discussion

Endometriosis is a prevalent chronic gynecological condition that can result in chronic pelvic pain, menstrual irregularities, and infertility, thereby significantly impacting women's reproductive health (22–24). The psychological burden associated with endometriosis, including anxiety and depression, is substantial (25, 26). Laparoscopic surgery is a common treatment for endometriosis cysts, and there is an elevated incidence of psychological issues among perioperative patients. Our research indicates that over half of the patients with endometriosis cysts experience anxiety and depression before surgery. Consequently, identifying a straightforward, non-invasive, and non-pharmacological intervention to ameliorate the perioperative psychological issues in these patients is of paramount importance.

Music listening interventions have the potential to alleviate stress by stimulating the parasympathetic nervous system, thereby reducing heart rate, blood pressure, and levels of stress hormones. This physiological response can lead to a significant reduction in postoperative pain and anxiety (13). In our previous randomized controlled trial, we observed that music listening interventions did not significantly alleviate postoperative pain and anxiety in young patients with nulliparous endometriosis cysts (15).

Endometriosis cysts can be categorized into unilateral and bilateral types, with the latter often presenting more complex conditions. Bilateral endometriosis is more correlated with deep infiltrating endometriosis, potentially complicating surgical management and elevating the risk of postoperative complications, including a decrease of anti-Müllerian hormone(AMH) (18, 19). Given the distinct nature of unilateral vs. bilateral endometriosis cysts, we conducted a secondary stratification analysis to investigate the postoperative effects of music listening interventions on patients with these conditions. Beyond examining the impacts on postoperative pain, anxiety, postoperative nausea, and vomiting, our study also incorporated additional indicators such as other psychological states, including depression and fatigue. Despite substantial theoretical evidence supporting the use of music as a non-pharmacological intervention for alleviating postoperative psychological and physiological distress (14, 21), our secondary analysis did not demonstrate any statistically significant benefits for patients undergoing laparoscopic cystectomy for unilateral or bilateral endometriotic cysts. In both subgroups, the music listening intervention did not result in meaningful improvements in postoperative pain (measured by the VAS), anxiety (assessed using the GAD-7 scale), depression (evaluated with the PHQ-9), fatigue (measured by the FSS), or nausea/vomiting, even when accounting for the increased disease burden and psychological vulnerability associated with bilateral cysts. These findings align with our initial randomized controlled trial, which also indicated the limited efficacy of music listening intervention in young, nulliparous patients with endometriosis.

The lack of observed benefits does not necessarily negate the therapeutic potential of music listening interventions. Several factors may account for these findings: (1) Significant variations exist in the impact of different music genres and intensities on both physical and psychological responses. Empirical evidence indicates that classical music is particularly effective in enhancing parasympathetic nerve activity (27). Additionally, music with varying tempos exerts distinct effects on stress and pain management (28, 29). Future research will aim to incorporate a broader range of music types and intensities in comparative studies to further investigate the non-pharmacological therapeutic potential of music listening interventions. (2) The music listening interventions were confined to a period of 7 days post-operation, with sessions lasting only 30 min per day. This level of intensity may be insufficient to produce a significant impact on postoperative psychological outcomes. Future research could investigate progressive dosing regimens, such as extended treatment durations or more frequent interventions, to ascertain whether threshold effects exist for therapeutic benefits. (3) Although patients selected music via a platform developed by professional therapists at our hospital, thereby ensuring their autonomy, the absence of direct engagement with well-trained music therapists may have weakened the intervention's efficacy. Professional music therapists can tailor music selections and guide patients in using music to regulate emotions more intentionally—skills that may enhance the therapeutic impact beyond self-selected listening alone. Supporting this, a previous study revealed that music therapy facilitated by music therapists served as a viable adjunct to standard gynecological surgical care, significantly reducing preoperative anxiety in women undergoing laparoscopic total hysterectomy for benign conditions (30). This suggests that structured guidance from specialists might be a critical factor in maximizing the benefits of music-based interventions, which our current self-administered protocol lacked. (4) Despite initial calculations ensuring adequate statistical power, the sample size became relatively small following stratified analysis, potentially compromising the statistical robustness of the results. Future studies will seek to increase the sample size to enhance statistical power and reliability.

Our study presents several implications for postoperative care, particularly concerning the integration of music listening interventions. It is important to note that while an initial observation suggested reduced anxiety among patients with bilateral cysts who received music listening interventions, this finding did not remain significant after correction for multiple comparisons. As such, it should be interpreted with caution as a preliminary trend rather than conclusive evidence of efficacy.

Considering that psychological distress can adversely affect recovery and overall patient satisfaction, the potential of music listening interventions to complement postoperative care protocols—when optimized—deserves further exploration to promote a more holistic and patient-centered approach. Nevertheless, the absence of consistent significant effects across outcomes indicates that music listening interventions should remain a complementary strategy rather than a replacement for conventional treatments within multimodal postoperative care.

This study represents the inaugural investigation into the application of music listening interventions for the postoperative management of endometrial cysts. Although definitive positive outcomes were limited, this research opens a novel pathway for exploring non-pharmacological interventions in addressing the postoperative psychological dimensions of endometriosis. Future research efforts will focus on validating the clinical efficacy of music listening interventions by expanding the sample size, extending the follow-up period, and conducting a comprehensive examination of the underlying mechanisms.

## Data Availability

The original contributions presented in the study are included in the article/Supplementary material, further inquiries can be directed to the corresponding author/s.
