# Supplementary material for: The effects of music listening intervention on postoperative care in unilateral vs. bilateral endometriotic cysts
Source: Front Med (Lausanne). 2025 Sep 15;12:1626575. doi: 10.3389/fmed.2025.1626575 (PMC12477193; doi:10.3389/fmed.2025.1626575)
Supplement: Supplementary file 1 [file Supplementary_file_1.pdf]

Music-based Interventions checklist

Item number		Item	Locationb (page or appendix number)
1		**Brief Name**<br>Provide the name or phrase that describes the intervention.	Music listening intervention on endometriotic cysts
2		**Intervention Theory and/or Scientific Rationale**<br>Provide a rationale for the music and/or music experience(s). Specify how essential features of the music and music experience(s) are expected to influence targeted outcomes.	Listening to music after surgery can lessen pain catastrophizing and may also enhance psychological and physical well-being during negative pain experiences. In the context of anxiety management, music has been empirically validated as a potent therapeutic intervention. Music distracts patients during medical procedures and reduces their perception of pain and anxiety-inducing stimuli. At the same time, it lowers the excitability of the sympathetic nervous system, slows down heart rate, and lowers blood pressure, thereby reducing anxiety. Furthermore, engaging with music has been shown to alleviate the severity of depressive symptoms and enhance sleep quality.
		**Intervention Content**<br>For Items 3a–3e, describe the music intervention with enough detail to support replication. When applicable, describe procedures for tailoring the intervention.	
	3a	**Music Selection**<br>Describe the process for how music was selected including who was involved in music selection	The patients chooses the music by themselves

3	3b	**Music**<br>Specify key details about the music that may be relevant to specified outcomes of interest. Characteristics may include compositional features of the music (such as tempo, harmony, rhythm, pitch, tonality, form, instrumentation),sound intensity or volume, lyrics, and/or how the music relates to the participants' cultural identity and heritage. When using published music, provide reference for a sound recording or sheet music.	Music selection was based on the "Music for Emotional Regulation" module within the "Cloud SanYuan" WeChat mini-program of the Third Affiliated Hospital of Sun Yat-sen University. This mini-program was developed by professional music therapists. The music library within the program is categorized based on therapeutic functions, emotional attributes, and musical characteristics, all defined according to principles of music therapy and clinical experience. The categories include: (1) Therapeutic function-based classifications: anxiety-relieving, depression-alleviating, and hypnotic music; (2) Emotion-or imagery-based classifications: emotional resonance, excitement, lightness, passion, quietness, beauty, and cheerfulness; (3) Acoustic characteristic-based classifications: low pitch, narration, and softness. To access the specific music program, please scan the following QR code on WeChat:

	3c	**Music Delivery Method**<br>Provide details about how music was provided to or created with participants (such as live, recorded, computer<br>generated).<br>Include any details necessary for replication. This might include size of performing group, use of playback equipment, or person controlling volume.	Speakers or headphones.
	3d	**Materials**<br>List all materials necessary for the music experience. Include music and non-music equipment and materials.	Music-related equipment<br>Personal smartphone (iOS or Android) with:<br>• WeChat app pre-installed<br>• Hospital "Music for Emotional Regulation" mini-program bookmarked<br>• Closed-back stereo headphones or speakers<br><br>Non-music equipment & materials<br>• Preoperative preparation and examination<br>• Surgical instruments<br>• General postoperative care materials
	3e	**Intervention Strategies**<br>Describe the music intervention strategy or strategies being studied (such as music listening, improvisation, song writing, rhythmic auditory stimulation)	1. Music intervention began one day before surgery and continued daily for seven days postoperatively, constituting one treatment course. Patients listened to preselected music at a comfortable volume for 30minutes daily, either through speakers or headphones.<br>2.Music selection was based on the "Music for Emotional Regulation" module within the "Cloud SanYuan" WeChat mini-program of the Third Affiliated

			Hospital of Sun Yat-sen University. A wide range of musical styles was available, allowing patients to choose their preferred type (fast and powerful, moderately paced and cheerful, or slow and soothing). Each session was limited to 30minutes.
4		**Interventionist**<br>Specify interventionist qualifications, credentials, training, and/or experience. Indicate how many interventionists delivered the music experience.	1. Physicians: All surgeons are senior physicians, including associate chief physicians and senior attending physicians.<br>2. Nurses in the inpatient department : Both preoperative and postoperative care are provided by senior nurses who offer general care, record various postoperative indicators, and guide the filling out of questionnaires.<br>3. Anesthesiologist: Senior anesthesiologists perform anesthesia and intraoperative monitoring.<br>4. Operating room Nurse: A professional nurse with a practice certificate.
5		**Individual or Group Intervention**<br>Specify whether interventions were delivered to individuals or groups of individuals. For group interventions, specify the size of the group.	Individual intervention
6		**Setting**<br>Describe where the intervention was delivered. Include location, privacy level, ambient sound, and/or any other factors that may have affected participants' experiences.	• Location: All participants were in-patients housed in two-bed hospital rooms.<br>• Delivery mode: Participants could choose headphones (primary option) or the room's built-in speaker at low volume.Most of sessions used personal, single-use headphones provided by the study.<br>• Safety protocol: Before each session, participants

		informed the ward nurse. A dedicated research nurse then documented start/end times and completion status on the study log sheet; no adverse events related to music listening were recorded.
7	Intervention Delivery Schedule<br>Report number of sessions, session length (for example, 60 min), frequency (for example, 3×/week), time interval between sessions (for example, single day, three consecutive days), and duration (for example, over 4 weeks).<br>Include practice, experiences, or tasks that are assigned to participants between intervention sessions.	Music intervention began one day before surgery and continued daily for seven days postoperatively, constituting one treatment course.<br>Listening sessions were self-selected at any time between 08:00–17:00; 30 minutes per day.
8	**Treatment Fidelity**<br>Describe strategies and/or measures used to ensure that the music intervention was delivered and received as intended.	The study was meticulously overseen and administered by a team of dedicated nurses to ensure timely completion of the music intervention and associated questionnaires by the participants. The study was subject to suspension and subsequent removal under the following circumstances: ① Non-adherence to the treatment protocol and inability to ascertain clinical efficacy; ② Incomplete clinical data that compromise the evaluation of efficacy and safety; ③ Participants exhibiting significant resistance during the clinical research process; ④ Discontinuation is warranted by clinical researchers, who are required to provide a rationale and conduct a treatment evaluation at the point of discontinuation.
